# Impact of viral presence in tumor on gene expression in non-small cell lung cancer

**DOI:** 10.1186/s12885-018-4748-0

**Published:** 2018-08-22

**Authors:** Youngchul Kim, Christine M. Pierce, Lary A. Robinson

**Affiliations:** 1 Department of Biostatistics and Bioinformatics, Moffitt Cancer Center, Tampa, 33612-9416 Florida USA; 2 Department of Cancer Epidemiology, Moffitt Cancer Center, Tampa, 33612-9416 Florida USA; 3 Center for Immunization and Infection Research in Cancer, Moffitt Cancer Center, Tampa, 33612-9416 Florida USA; 4 Division of Thoracic Oncology, Moffitt Cancer Center, Tampa, Florida 33612-9416 USA

**Keywords:** Non-small cell lung cancer, Virus, Gene expression, Carcinogenesis, Retrovirus, Human papilloma virus, HPV

## Abstract

**Background:**

In our recent study, most non-small-lung cancer (NSCLC) tumor specimens harbored viral DNA but it was absent in non-neoplastic lung. However, their targets and roles in the tumor cells remain poorly understood. We analyzed gene expression microarrays to identify genes and pathways differentially altered between virus-infected and uninfected NSCLC tumors.

**Methods:**

Gene expression microarrays of 30 primary and 9 metastatic NSCLC patients were preprocessed through a series of quality control analyses. Linear Models for Microarray Analysis and Gene Set Enrichment Analysis were used to assess differential expression.

**Results:**

Various genes and gene sets had significantly altered expressions between virus-infected and uninfected NSCLC tumors. Notably, 22 genes on the viral carcinogenesis pathway were significantly overexpressed in virus-infected primary tumors, along with three oncogenic gene sets. A total of 12 genes, as well as seven oncogenic and 133 immunologic gene sets, were differentially altered in squamous cell carcinomas, depending on the virus. In adenocarcinoma, 14 differentially expressed genes (DEGs) were identified, but no oncogenic and immunogenic gene sets were significantly altered. In bronchioloalveolar carcinoma, several genes were highly overexpressed in virus-infected specimens, but not statistically significant. Only five of 69 DEGs (7.2%) from metastatic tumor analysis overlapped with 1527 DEGs from the primary tumor analysis, indicating differences in host cellular targets and the viral impact between primary and metastatic NSCLC.

**Conclusions:**

The differentially expressed genes and gene sets were distinctive among infected viral types, histological subtypes, and metastatic disease status of NSCLC. These results support the hypothesis that tumor viruses play a role in NSCLC by regulating host genes in tumor cells during NSCLC differentiation and progression.

**Electronic supplementary material:**

The online version of this article (10.1186/s12885-018-4748-0) contains supplementary material, which is available to authorized users.

## Background

Viruses and other infectious agents cause nearly 20% of all human cancers worldwide such as human papillomavirus (HPV) in cervical carcinoma and hepatitis B virus (HBV) in hepatocellular carcinoma [1]. There is growing evidence that viruses play a critical role in cancer development as well as modulating the response to cancer treatment [2].

Using advanced panmicrobial microarray techniques with polymerase chain reaction (PCR) confirmation in our recent study, we searched for viral DNA sequences in archived frozen non-small cell lung cancer (NSCLC) tumor of various cell types [3]. We found that the majority of NSCLC tumor samples contained viral DNA sequences from ten viral types including exogenous retroviruses and HPV while no viral DNA was detected in any adjacent non-neoplastic lung tissue samples. We also discovered that the susceptibility of lung cancer to viral infection generally varied across its cell types and by the types of viruses, suggesting that lung cancer subtypes could be associated with viral types residing in host cells. Interestingly, NSCLC patients with viral DNA present in their tumors had significantly longer overall survival than those not containing viral DNA.

However, the impact of viruses on host NSCLC tumor cells remains poorly understood and only few studies to date have investigated the roles of virus in the NSCLC [4]. Viruses can cause cellular transformation by expression of viral oncogenes, by genomic integration to alter the activity of cellular proto-oncogenes or tumor suppressor genes and by inducing inflammation that promotes oncogene activity [5]. We therefore hypothesized that viruses in human lung cancer cells have the potential ability to affect host cells by regulating expression levels of important genes, especially oncogenes and immune-related genes.

We expanded our previous analysis [3] to better understand the association of viral infections with host gene expressions in the NSCLC tumors by performing an extensive differential expression analysis of high-throughput gene expression profiling microarray data from the same fresh frozen archived NSCLC tumor specimens according to viral types, histological NSCLC subtypes, and metastatic disease status.

Clarification of target genes of viruses in human NSCLC tumor cells and the functions of the target genes will provide an opportunity to develop new prognostic and early diagnostic biomarkers of NSCLC as well as potential cancer prevention strategies.

## Methods

### Patients and microarray data

Florida residents who underwent surgical resection for NSCLC at Moffitt Cancer Center and consented to the Total Cancer Care protocol between 2000 and 2013 were eligible in our previous study for a viral DNA detection in NSCLC tumor tissue samples [3]. Approval for the use of archived tissue and patient information was obtained from the University of South Florida IRB, Protocol No. MCC16765.

We randomly selected 70 archived frozen NSCLC tumor samples based on: 1) having enough volume of frozen tissue to perform the studies, 2) preoperative radiographs showed no pneumonia or distal atelectasis, and 3) no patient had chemotherapy or radiotherapy before resection. Resulting NSCLC samples encompass 10 primary adenocarcinomas, 10 bronchioloalveolar carcinomas (BAC, although currently termed adenocarcinomas with lepidic spread) and 30 squamous cell carcinomas (SCCs). In addition, we selected 10 resected stage IV tumors (three SCCs and seven adenocarcinomas) and their 10 matched surgically-resected distant oligometastases. Anatomic sites of those 10 metastatic tumor specimens were brain (*n* = 3), soft tissue (*n* = 2), adrenal (*n* = 4) and kidney (*n* = 1). 10 non-neoplastic lung specimens were also obtained as controls for this study.

All primary and metastatic tumor specimens underwent viral DNA screening tests by the Lawrence Livermore PanMicrobial Detection Array (LLMDA) designed to detect all sequenced viral families. The LLMDA was developed at the Lawrence Livermore National Laboratory (LLNL; Livermore, CA, USA) and designed to detect all sequenced viral and bacterial families, with appropriate controls [6, 7]. The 135 K format of the LLMDA (v.5) targets all vertebrate pathogens including 1856 viruses, 1398 bacteria, 125 archaea, 48 fungi, and 94 protozoa [8]. In the development of this microarray, PCR was used extensively to validate the results and verify that the statistical algorithm was accurate [9–11]. The high-density oligo LLMDA and statistical analysis method has been extensively tested in numerous problems in viral and bacterial detection from pure or complex environmental or clinical samples [9, 12–15]. A subset of NSCLC tumors [10 squamous cell carcinomas (SCCs)] was evaluated using an oncovirus panel of the International Agency for Research on Cancer (46 HPV types, 10 polyomaviruses, and 5 herpesviruses) [16]. In addition, all 70 NSCLC underwent HPV PCR genotyping using the INNO-LiPA Genotyping Extra Assay which detects 28 HPV genotypes classified as high or low risk, depending on their association with carcinogenesis. Details concerning the detection techniques, patient’s clinical characteristics and virus DNA detection results can be found in our previous study [3]. For the current study, the Moffitt institutional honest broker retrieved 39 available gene expression microarrays from these same tumor samples, of which the platform was Rosetta/Merck Human RSTA Custom Affymetrix Genechip with 60,607 probe sets interrogating 25,285 genes.

### Data analysis

All microarray data were normalized using the iterative rank-order normalization algorithm [17], and experimental batch effects and outlying observations were further examined by a two-way hierarchical cluster analysis based on sample-wise correlation matrix as a distance matrix and a principal component cluster analysis. Unsupervised and supervised approaches for differential gene expression analysis and interpretation of resultant genes were utilized to associate virus infection with gene expressions in lung tumor cells according to NSCLC histological subtypes and metastasis status to minimize their potential confounding effects.

The unsupervised approach first used Linear Models for Microarray Analysis (LIMMA) to identify individual genes with significant differential expression between virus-infected and uninfected tumor samples [18]. Thereafter, a functional annotation and gene ontology (GO) analysis of the genes was performed by using Database for Annotation, Visualization and Integrated Discovery (DAVID) [19] and a database of virus-host protein-protein interactions (VirusMentha) [20].

For the supervised approach, Gene Set Enrichment Analysis (GSEA) was utilized to determine whether genes on a known biological or functional pathway have jointly concordant differential expressions between virus-infected and uninfected lung tumor specimens. Fifty hallmark, 189 oncogenic, and 4872 immunologic gene sets annotated in Molecular Signatures Database were subjected to GSEA [21]. For a multiple testing correction, false discovery rate (FDR)-adjusted *p*-values were estimated in all LIMMA, GO, and GSEA analyses, and a statistical significance was defined when the FDR-adjusted *p*-value was less than 0.2.

## Results

### Quality control analysis of gene expression microarrays

Sample-wise Spearman’s rank correlation coefficients of 39 microarrays over all probe sets ranged from 0.866 to 0.944. These high correlation coefficients indicated that a majority of genes had similar expression patterns across NSCLC tumor tissue samples regardless of their diverse histological subtypes and disease progression status. However, a two-way hierarchical cluster analysis and a principal component analysis of all microarrays revealed that all three brain metastatic NSCLC tumor tissue specimens (one SCC sample harboring the Y73 sarcoma virus (Y73SV) DNA, one uninfected SCC, and one uninfected adenocarcinoma samples) were clustered far apart from other primary lung tumor samples and the non-brain metastatic tumor samples, showing a brain-specific biological variation independent of viral infection status in gene expression profiles. We therefore excluded these three brain metastatic tumor samples from a differential gene expression analysis.

### Differentially expressed genes and pathways between all NSCLC with and without any viral DNA

**Fig. 1:**
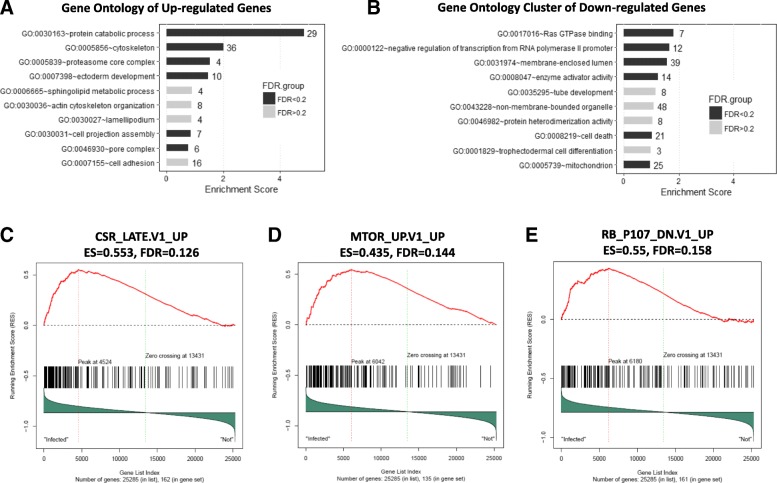
Gene Ontology Clusters of Differentially-Expressed Genes in all Primary and Metastatic NSCLC. **a**, **b**, Biological processes of overexpressed (**a**) and underexpressed genes (**b**) in Virus-infected NSCLC tumors, as compared to uninfected NSCLC tumors, were displayed. Black bars represent significantly overrepresented functions (FDR < 0.2). The number at the end of each bar indicates how many genes have the corresponding biological function. **c**, **d**, and **e**, Gene Set Enrichment Score were displayed for three significantly enriched oncogenic gene sets (FDR < 0.2). Positive enrichment score (ES) means that a majority of genes in those gene sets were concordantly overexpressed in Virus(+) NSCLC tumors

To identify differentially expressed genes (DEGs), LIMMA analysis was applied to a total of 36 NSCLC tumor samples: 21 samples harboring viral DNA of at least one viral type (Virus(+)) and 15 samples without any viral DNA (Virus(−)). This analysis identified 338 overexpressed and 301 underexpressed genes in Virus(+), as compared to Virus(−) (FDR *p* < 0.2; Additional file 1**:** Figure S1). For instance, PCYT1A was the most significantly overexpressed gene in Virus(+) (fold change (FC) = 2.24) whereas JMJD1C and CTNNB1 were the top two underexpressed genes with FC of 0.44 and 0.45, respectively. (Additional file 2: Table S1). Li et al. (2015) reported that PCYT1A catalyzes the rate-limiting step in synthesis of phosphatidylcholine that is required for replication of HBV. They also confirmed that PCYT1 was up-regulated at the transcriptional level in HBV-infected human hepatoblastoma cells [22]. In addition, Vaezi et al. (2014) found that PCYT1A is the dominant determinant of 8F1 immunoreactivity in lung SCC samples and that high expression of PCYT1A was found to be prognostic of longer disease-free survival [23]. JMJD1C is a component of DNA-damage response pathway with implications for tumorigenesis by demethylating MDC1 to regulate the RNF8 and BRCA1-mediated chromatin response to DNA breaks. Chen et al. (2016) identified that the gain of the miRNA regulation of MIR141 to JMJD1C resulted in the gain of protein-protein interaction between JMJD1C and GADD45A in hepatocarcinogenesis that is a multistep process mainly associated with persistent infection with HBV or hepatitis C virus [24]. CTNNB1 is considered the cancer drivers for hepatocellular carcinoma development with variable frequencies depending on the etiology. A recent genome-wise RNAi screen revealed that a role of a WNT/CTNNB1 signaling pathway as negative regulator of virus-induced innate immune responses [25]. Nakayama et al. (2014) also reported that pharmacological inhibition or conditional deletion of CTNNB1 inhibited lung tumor formation in transgenic mice [26]. A functional annotation of those DEGs was performed using DAVID tool to gain insight into their biological functions. For the 338 overexpressed genes in Virus(+), the protein catabolic process was the most significantly over-represented function (29 hit genes, *p* < 0.001) followed by cytoskeleton and proteasome core complex. For the 301 underexpressed genes in Virus(+), six biological processes, such as Ras GTPase binding and RNA polymerase II promoter, were significantly enriched (Fig. 1a and b). GSEA was next performed to identify predefined sets of hallmark, oncogenic, and immunologic genes having concordantly differential expressions between the Virus(+) and Virus(−) NSCLC samples. As a result, three oncogenic gene sets, CSR_late.v1.up, mTOR_up.v1_up, Rb_P107_dn.v1_up, were found to be significantly altered with positive enrichment scores, meaning that a majority of genes in those gene sets were simultaneously overexpressed in the Virus(+) **(**Fig. 1c, d, and e**)**. Among them, the CSR_late_up.v1_up gene set comprises 172 genes up-regulated in late serum response of human foreskin fibroblasts and associated with increased risk of metastasis and death in human lung, breast, and gastric cancer [27].

### All primary NSCLCs: virus carcinogenesis and oncogenic gene sets enriched in virus-infected specimens

**Fig. 2:**
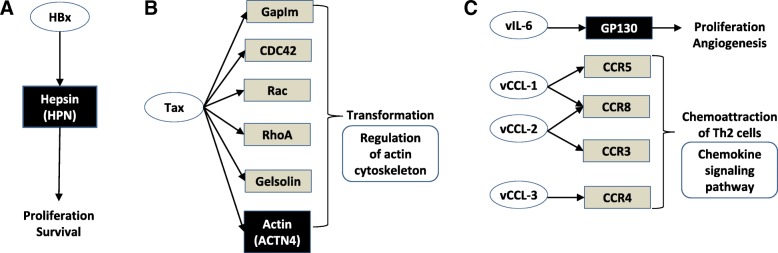
Viral carcinogenesis pathway and differentially expressed genes in primary NSCLC tumors. The viral carcinogenesis was displayed in part, focusing on three over-expressed genes in virus-infected Primary NSCLC tumors in comparison with uninfected primary tumors. Eclipse and rectangle boxes indicate viral product and host cellular target, respectively. **a** HPN (hepsin), **b** ACTN4 (actinin alpha 4), and **c** GP130 (interleukin 6 signal transducer) are host cellular targets of three different viral oncoproteins (HBx, Tax, and vIL-6) that lead to cell proliferation/survival, regulation of actin cytoskeleton, and proliferation angiogenesis, respectively

LIMMA was performed to identify DEGs between 20 virus-infected primary NSCLC tumor specimens (Virus(+)) and 10 uninfected specimens (Virus(−)). Seven hundred seventy-seven genes were significantly overexpressed in Virus(+) and 751 genes underexpressed (Additional file 3: Figure S2). To understand biological meaning behind these DEGs and discover enriched functional-related gene groups, a functional annotation enrichment analysis was performed using the DAVID tool. Table 1 showed their functional annotation results. For the overexpressed genes, the cell cycle was the most significantly overrepresented function (23 hit genes, *p* < 0.001). Strikingly, two virus-related biological processes, 1) viral carcinogenesis (22 hit genes) and 2) human T-cell lymphotropic virus Type I (HTLV-1) infection (23 hit genes), were also significantly overrepresented, along with several NSCLC tumorigenesis-related pathways, including proteasome pathway [28] and p53 signaling pathway [29]. In particular, HPN (hepsin), ACTN4 (actinin alpha 4), and GP130 (interleukin 6 signal transducer) on the viral carcinogenesis pathway were known to be host cellular targets of three viral oncoproteins (HBx, Tax, and vIL-6) that lead to cell proliferation/survival, regulation of actin cytoskeleton, and proliferation angiogenesis, respectively (Fig. 2**;** Additional file 4: Figure S3) [30–32]. On the other hand, cAMP signaling, vascular smooth muscle contraction, and metabolic pathways were the most representative pathways for the underexpressed genes in Virus(+) (FDR *p* < 0.05). A recent study reported that the cAMP signaling augments radiation-induced apoptosis in NSCLC cells [33].

**Table 1:** Overrepresented KEGG pathways of differentially expressed genes in virus-infected versus uninfected primary NSCLC specimens

	KEGG ID	Name	Count	Pop.Hits	Fold Enrichment	FDR	Genes	Total list
Over-expressed genes in Virus-infected primary NSCLC tumors	hsa04110	Cell cycle	23	124	4.121	0.000	E2F2, CDC6, FZR1, E2F4, RBL1, SKP2, PKMYT1, CHEK1, CDC20, PTTG1, MCM2, CDK4, CDC25B, MCM6, CCNE1, CDC45, CCNB2, CCND2, TFDP2, BUB1, BUB1B, CCNA2, STAG1	311
	hsa03050	Proteasome	10	44	5.050	0.001	PSMF1, PSMC4, PSMC3, PSMD11, PSME2, PSMB3, PSMB2, PSMD2, PSMD3, PSME3	311
	hsa04115	p53 signaling pathway	12	67	3.979	0.002	CCNE1, CCNB2, CCND2, SERPINB5, RRM2, BAX, CHEK1, PMAIP1, PERP, CDK4, GTSE1, SESN3	311
	hsa05203	Viral carcinogenesis	22	205	2.384	0.004	HRAS, RBL1, UBR4, SKP2, ACTN1, CHEK1, CDC20, PMAIP1, MAPKAPK2, CDK4, SRF, PKM, CDC42, CCNE1, MAPK1, GTF2A1, CCND2, BAX, RANBP1, CCNA2, CHD4, DLG1	311
	hsa04120	Ubiquitin mediated proteolysis	16	137	2.595	0.015	FZR1, UBE2A, SOCS1, CBL, UBE4B, SKP2, UBE2J1, SAE1, CDC20, KEAP1, UBE2L3, BRCA1, FANCL, PIAS2, PIAS1, UBE2S	311
	hsa03015	mRNA surveillance pathway	12	91	2.930	0.030	NXT1, NCBP2, SYMPK, FIP1L1, ALYREF, HBS1L, SRRM1, MSI2, SMG1, ETF1, PPP2R2B, PPP2R3C	311
	hsa05166	HTLV-I infection	23	256	1.996	0.031	DVL3, E2F2, IL6, HRAS, TLN2, SLC25A5, ELK1, CHEK1, CDC20, PTTG1, CD40, MYBL2, CDK4, SRF, MSX2, POLE2, ELK4, CCND2, BAX, SLC2A1, BUB1B, RANBP1, DLG1	311
	hsa03040	Spliceosome	13	133	2.172	0.187	NCBP2, DHX8, SNRPA1, TRA2B, LSM6, U2SURP, ALYREF, SF3B3, CTNNBL1, SRSF4, TCERG1, SNRNP40, SNRPF	311
Under-expressed genes in Virus-infected primary NSCLC tumors	hsa04024	cAMP signaling pathway	18	198	2.804	0.003	ACOX1, ATP1B1, ROCK1, ROCK2, ADCY6, PDE4D, PDE4C, ATP1A2, PPP1CB, PLCE1, GRIA1, ABCC4, RAP1A, HHIP, HCN4, ADCY10, CACNA1D, HCAR1	224
	hsa04270	Vascular smooth muscle contraction	13	119	3.370	0.005	ROCK1, PLA2G10, ROCK2, PPP1R12B, ADCY6, NPR2, ARHGEF12, PPP1CB, GNAQ, PLA2G12A, PLA2G12B, CACNA1D, PPP1R14A	224
	hsa01100	Metabolic pathways	59	1228	1.482	0.015	ACOX2, ACOX1, CYP3A5, COX11, SGMS2, AMT, ALG2, ADH1A, PPOX, GPAT2, HIBADH, ASAH1, PDHB, ASAH2, ASPA, PIGM, NDUFS8, BPNT1, AGPAT2, NDUFS1, COX15, IDUA, NMNAT2, C1GALT1C1, HMGCLL1, SUCLG2, COX4I2, LPIN2, CDS1, TAT, ALDH3B1, ATP6V1C1, PLCE1, ALOX15B, MGAT5, AOC1, PRODH, ME3, LOC102724788, ALDOB, CTPS2, PLA2G12A, PLCH1, B3GNT6, PLA2G12B, BDH2, HSD17B7, ACSL5, PLA2G10, B3GALT2, KL, MAOA, NAT1, ACSM3, DBT, PON2, AHCYL2, ABO, PON3	224
	hsa04390	Hippo signaling pathway	12	151	2.452	0.114	BMP4, PARD6B, BMP2, TP53BP2, WTIP, FZD5, PPP1CB, BMP5, LLGL2, BMPR1A, CTNNB1, PPP2R2A	224
	hsa04972	Pancreatic secretion	9	93	2.985	0.121	ATP1B1, SLC12A2, GNAQ, PLA2G10, PLA2G12A, PLA2G12B, ADCY6, RAP1A, ATP1A2	224
	hsa00564	Glycerophospholipid metabolism	9	95	2.922	0.135	GPD1L, LPGAT1, PLA2G10, PLA2G12A, PLA2G12B, LPIN2, GPAT2, CDS1, AGPAT2	224
	hsa04961	Endocrine and other factor-regulated calcium reabsorption	6	45	4.113	0.168	ATP1B1, GNAQ, KL, PTH1R, ADCY6, ATP1A2	224
	hsa04510	Focal adhesion	14	206	2.096	0.182	COL4A4, COL4A3, ROCK1, PAK3, ROCK2, FLT4, PPP1R12B, ITGA8, ITGA1, ITGA10, RAP1A, ACTN2, PPP1CB, CTNNB1	224
	hsa04146	Peroxisome	8	83	2.973	0.199	ACOX2, ACOX1, HMGCLL1, NUDT12, PEX1, ABCD3, PEX13, ACSL5	224

Count: the number of genes on the corresponding KEGG pathway among input DEGs;

Pop Hits: the number of genes on the corresponding KEGG pathway among all human genes

FDR: false discovery rate–adjusted *p*-value

Total list: the number of input DEGs

Using GSEA of hallmark gene sets, e2F transcription factor target, G2/M-checkpoint, mTORC1 signaling, and mitotic spindle assembly were found to be concordantly over-expressed gene sets in Virus(+) (all FDR *p* < 0.2; Additional file 5: Figure S4A). The GSEA analysis of 189 oncogenic gene sets also revealed that Rb/P107_DN.v1.up, Rb_down [34], e2F1 target [35], GCNP/SHH [36], and mTOR_up.v1_up [37] were significantly enriched again with all positive enrichment scores (all FDR *p* < 0.2; Additional file 5: Figure S4B**)**.

### Primary squamous cell carcinoma: differentially expressed genes varied depending on infection viral types

Noticeably, all SCC tumor specimens bear viral DNA of at least one viral type and thus differential gene expression analyses were performed for each viral type except HBV, with which only one SCC specimen was uninfected. Table 2 shows the list of 12 DEGs from the LIMMA analysis of the SCC specimens with and without viral DNA of each of Bovine leukemia virus (BLV), *Panthera leo persica* Papillomavirus Type 1 (PlpPV1), HPV, Simian T-cell leukemia virus Type 1 (STLV1), Type 2 (STLV2) and Type 6 (STLV6).

**Table 2:** Differentially expressed genes in virus-infected versus uninfected primary NSCLC subtypes

NSCLC Subtype	Virus (the number of tumors infected)	Gene Symbol	Average Expression when in Virus-uninfected	Average Expression in Virus-Infected	Fold Change	Up/Down in Virus-infected tumors	FDR q-value	Gene Description
Squamous cell carcinoma (*n* = 10)	BLV (*n* = 8)	PSG4	58.325	6.142	0.105	down	0.033	pregnancy specific beta-1-glycoprotein 4
		CPB2	124.383	8.635	0.069	down	0.156	carboxypeptidase B2
	PlpPV.1 (*n* = 2)	GCM1	7.393	39.660	5.365	up	0.067	glial cells missing homolog 1
		SMR3A	9.305	86.279	9.273	up	0.099	submaxillary gland androgen regulated protein 3A
	STLV.1 (*n* = 2)	MYEOV	8.732	60.041	6.876	up	0.048	myeloma overexpressed
		SPRR3	3205.815	15.053	0.005	down	0.048	small proline rich protein 3
		FMN2	13.410	503.286	37.532	up	0.056	formin 2
		GATA4	8.371	44.251	5.287	up	0.074	GATA binding protein 4
		LOC105375229	8.548	87.248	10.207	up	0.120	uncharacterized LOC105375229
		SEMA5B	70.639	302.948	4.289	up	0.120	semaphorin 5B
		CNTNAP4	7.677	44.480	5.794	up	0.197	contactin associated protein like 4
	STLV.2 (*n* = 3)	CRISP2	7.371	91.073	12.355	up	0.015	cysteine rich secretory protein 2
Adenocarcinoma (*n* = 10)	Any (*n* = 8)	ACTC1	31.21	6.37	0.20	down	0.187	actin, alpha, cardiac muscle 1
		PCSK2	260.71	19.03	0.07	down	0.144	proprotein convertase subtilisin/kexin type 2
	HBV (*n* = 2)	CEACAM8	6.00	226.44	37.74	up	0.000	carcinoembryonic antigen related cell adhesion molecule 8
		PRSS1	9.37	238.98	25.49	up	0.035	protease, serine 1
		CALB2	12.70	244.70	19.27	up	0.056	calbindin 2
		NUDT4	6.41	25.36	3.96	up	0.109	nudix hydrolase 4
		NAP1L6	5.57	19.92	3.57	up	0.071	nucleosome assembly protein 1 like 6
		CEP170B	192.56	68.38	0.36	down	0.083	centrosomal protein 170B
		MTND6P4	5241.96	1570.27	0.30	down	0.109	mitochondrially encoded NADH:ubiquinone oxidoreductase core subunit 6 pseudogene 4
		NUP62	1710.30	406.58	0.24	down	0.012	nucleoporin 62 kDa
	Y73SV (*n* = 6)	MARCH3	14.08	36.38	2.58	up	0.151	membrane associated ring-CH-type finger 3
		ANKRD29	32.33	9.34	0.29	down	0.151	ankyrin repeat domain 29
		NMNAT2	70.41	11.09	0.16	down	0.151	nicotinamide nucleotide adenylyltransferase 2
		QSER1	386.06	47.97	0.12	down	0.151	glutamine and serine rich 1

For BLV, PSG4 and CPB2 were significantly underexpressed in BLV(+) (*n* = 8), as compared to BLV(−) SCC specimens (*n* = 2). Of these, CPB2 is an extracellular matrix-regulated gene and has been considered an indicator for an impaired lung function [38] (Additional file 6: Figure S5A).

**Fig. 3:**
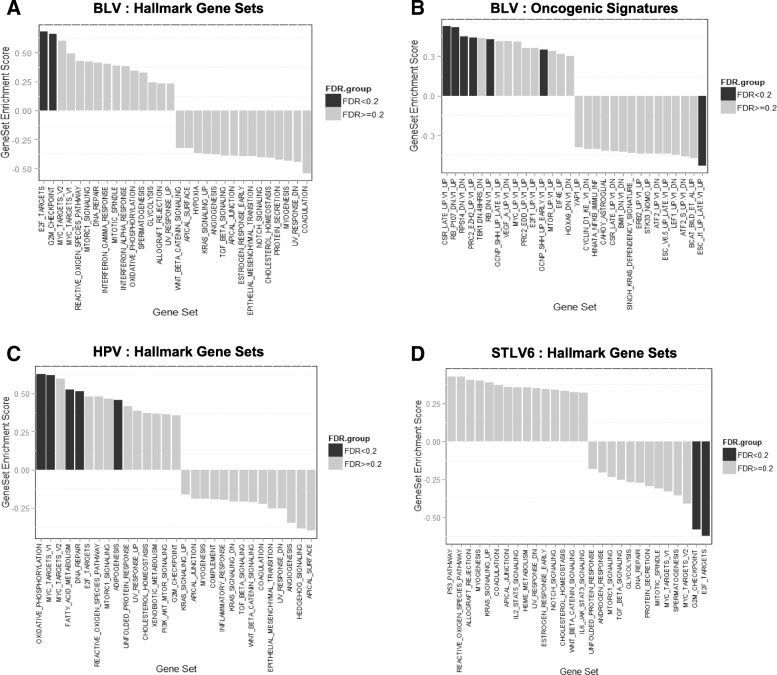
Gene Sets Enriched Significantly in Primary Squamous Cell Carcinoma. GSEA was performed on hall mark, oncogenic, and immunologic gene sets for individual viral type detected in primary squamous cell carcinoma. A positive GSEA score indicates that a majority of genes in the corresponding gene set are concordantly overexpressed in virus-infected SCC specimens and vice versa. Black bars represent significantly enriched gene sets (FDR < 0.2). **a** hallmark gene sets compared between BLV(+) and BLV(-) primary SCC **b** oncogenetic gene sets compared between BLV(+) and BLV (-) primary SCC, **c** hallmark gene sets compared between HPV(+) and HPV(-) primary SCC; **d** hallmark gene sets compared between STLV(+) and STLV(-) primary SCC

The GSEA of hallmark gene sets resulted in two significantly altered cell cycle-pathways. One was the G2/M checkpoint (FDR = 0.019) and the other was the e2F targets that encode cell cycle-related targets of e2F transcription factors (FDR *p* = 0.054) (Fig. 3a). Successively, in the GSEA of oncogenic gene sets, seven significant signatures came up and the most significant signature was a set of genes up-regulated in primary keratinocytes with knockout of both Rb1 and Rbl1 (FDR *p* = 0.038) (Fig. 3b) [34]. Above all, the GSEA of immunologic gene sets revealed that 133 immunologic signatures were significantly enriched. Among these, the top significant signature was WT_vs_NFATC1_KO, which comprises 200 genes up-regulated in B lymphocytes stimulated by anti-IgM under knockout of NFATc1 (ES = 0.674, FDR *p* = 0.026; Additional file 6: Figure S5B). This suggested that BLV in SCC tumor cells might interact closely with NFATc1, which is an oncogene involved in various functions in cancer [39, 40].

For PlpPV1, GCM1 and SMR3A genes were significantly overexpressed in PlpPV1(+) (*n* = 2) in comparison with PlpPV1(−) specimens (*n* = 8). As for STLV1, comparisons of gene expression between STLV1(+) (*n* = 2) and STLV1(−) specimens (*n* = 8) yielded five significantly overexpressed, such as FMN2 and MYEOV, and one down-regulated gene (SPRR3) in STLV1(+). A comparison of gene expressions between STLV2(+) (*n* = 3) and STLV2(−) specimens (*n* = 7) detected only one gene, CRISP2 (cysteine rich secretory protein 2), that was significantly down-regulated in STLV2(+) (Table 2).

Interestingly, although the LIMMA analysis for HPV57 and STLV6 viral types resulted in no significant DEG, GSEA yielded several significantly enriched gene sets. For HPV57, five hallmark gene sets, such as oxidative phosphorylation and Myc targets, were significantly enriched (Fig. 3C). Additionally, it was a unique oncogenic gene set that TBK1.DN.48 enriched significantly **(**ES = 0.58, *p* = 0.049; Additional file 6: Figure S5C). This gene set comprises 50 genes down-regulated in epithelial lung cancer cells upon over-expression of the proto-oncogene KRAS and knockdown of TBK1, and induced apoptosis [41].

Lastly, the GSEA of hallmark gene sets for comparing STLV6(+) (*n* = 3) with STLV6(−) specimens (*n* = 7) recaptured G2/M checkpoint (ES = − 0.581, FDR *p* = 0.053) and e2F transcription factor target (ES = − 0.622, FDR *p* = 0.084), both of which were also significantly altered in the aforementioned comparison between BLV(+) and BLV(−), but showed reversed expression changes with negative ESs, which seemed obvious because 6 of 7 STLV(−) SCC specimens were BLV(+) (Fig. 3d).

### Primary adenocarcinoma: No enrichment of oncogenic and immunologic gene sets

Eight of 10 (80%) primary adenocarcinoma specimens bear viral DNA of four different viral types (four with Y73SV only, two with HBV only, one with both HPV57 and Y73SV, and one with both Y73SV and STLV2). In the LIMMA analysis of any virus-infected (Virus(+)) (*n* = 8) versus uninfected primary adenocarcinoma specimens (Virus(−)) (*n* = 2), ACTC1 and PCSK2 were found to be significantly down-regulated in Virus(+) (Table 2**;** Additional file 7: Figure S6). ACTC1 is a member of actin cytoskeletons and was recently reported to be a potential candidate contributing to the enhanced lung tumor development [42].

A viral type-specific subgroup analysis was subsequently performed using the LIMMA for each of HBV and Y73SV with at least two infected samples. When HBV(+)(*n* = 2) and HBV(−) specimens (*n* = 8) were compared, five genes (CEACAM8, PRSS1, CALB2, NUDT4, and NAP1L6) were significantly over-expressed in HBV(+) whereas CEP170B, MTND6P4 and NUP62 were down-regulated (Table 2). Of these, PRSS1, CALB2, and NUDT4 shared a common molecular function of metal ion binding according to a DAVID gene ontology analysis. Noticeably, VirusMetha interactome analysis of the genes revealed that NUP62 protein has interactions with three viral proteins: P0C206, Q85601, and TAX. Additionally, NUP62 is an essential component of the nuclear pore complex and plays a novel role in centrosome integrity. A recent study noted that knockdown of NUP62 induced G2/M phase arrest, mitotic cell death, aberrant centrosome, and centriole formation [43].

Next the LIMMA analysis of six Y73SV(+) and four Y73SV(−) adenocarcinoma specimens identified one up-regulated gene, MARCH3, and three down-regulated genes, NMNAT2, ANKRD29, and QSER1, in Y73SV(+) specimens. NMNAT2 is involved in nicotinate and nicotinamide metabolism, and is a novel regulator of cell proliferation and apoptosis in NSCLC by binding with SIRT3 [44].

Unlike the GSEA results of SCC specimen data, however, no oncogenic and immunologic gene set was enriched in the GSEA analysis of primary adenocarcinoma data, indicating that viruses in SCC tumors might interact with human genes more strongly than those in adenocarcinoma.

### Primary bronchioloalveolar carcinoma: low virus infection rate

There were only two BAC specimens bearing viral DNAs of Porcine circovirus type 2 (PCV-2) and Y73SV, exclusively. Several immune-related genes had high expression levels in the both virus-infected specimens, but not statistically significant (e.g. IGKV1–5 with FC = 96 and FDR *p* = 0.47; IGKV1D-13 with FC = 228 and *p* = 0.99) (Additional file 8: Figure S7A). Likewise, no gene set was enriched in the GSEA even though notch-signaling hallmark gene set and CRX_NRL oncogenic signatures were highly overexpressed in the two virus-infected specimens (Additional file 8: Figure S7B and S7C).

### Metastatic lung cancer: distinct genes from the findings of primary tumor analysis

Y73SV was the unique viral type detected in two of nine metastatic tumor specimens. Therefore, all differential gene expression analyses were performed over all histological subtypes and metastatic disease sites. Using LIMMA, 69 DEGs including CRCT1 and MAGE9 were found (Additional file 9: Figure S8A). Based on DAVID annotation results of the DEGs, the top represented biological process was the positive regulation or activation of cell proliferation (FDR *p* = 0.13). It involved four overexpressed genes, BCL6, NTN1, NAMPT, and PBX1, and two underexpressed genes, MVD and VEGFC, in Y73SV(+) metastatic NSCLC specimens. The melanoma associated tumor antigen (MAGE) pathway was also significantly over-represented biological process and encompasses three genes (MAGEA9, MAGEA9B, and MAGEB2) overexpressed in Y73SV(+). Furthermore, 39 down-regulated genes were in part associated with the zinc finger binding process as well as the integral component of membrane (Additional file 9: Figure S8B, C, and D).

**Fig. 4:**
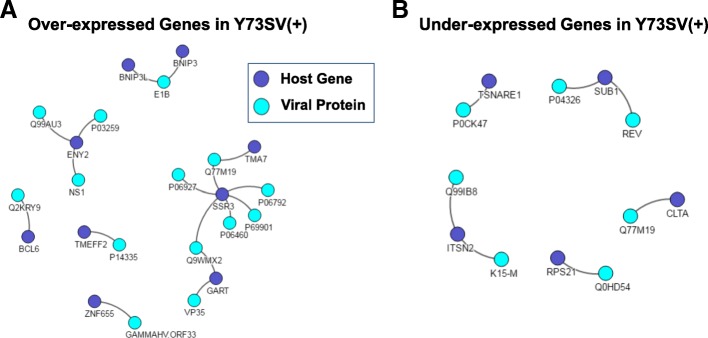
Virus-host protein-protein Interaction of differentially-expressed genes in virus-infected metastatic lung tumors. VirusMentha interactome analysis results are depicted for **a** nine overexpressed genes and **b** five under-expressed genes in Y73SV(+), as compared to Y73SV(-) metastatic lung tumor specimens. Viral proteins and their interaction target genes in host cells are colored cyan and indigo, respectively

The VirusMentha interactome analysis next revealed that nine over-expressed genes (BNIP3, BNIP3L, ENY2, BCL6, TMEFF2, ZNF655, TMA7, SSR3, and GART) and five under-expressed genes (CLTA, RPS21, SUB1, ITSN2, and TSNARE1) in Y73SV(+) had significant virus-host protein-protein interaction with human adenovirus 2 (FDR *p* = 0.019) and human immunodeficiency virus type 1 (FDR *p* = 0.027), respectively (Fig. 4). Among them, SSR3 has the most complex interaction network with viral proteins, in terms of the number of interacted viral proteins, followed by ENY2 and GART. In particular, BNIP3 is an apoptosis-inducing protein that can overcome Bcl-2 suppression and plays an important role in the calprotectin (S100A8/A9)-induced cell death pathway [45]. Both BNIP3 and BNIP3L interact with small T-antigen E1B viral protein that is a putative adenovirus Bcl-2 homolog that inhibits apoptosis induced by TNF or FAS pathways, as well as p53-mediated apoptosis [46]. It was also reported that without E1B function, virus production is compromised because of premature death of the host cell [47].

Lastly, in order to explore metastasis-specific genes, we compared the 69 DEGs with the 1527 DEGs, which was identified in the above primary tumor analysis comparing Virus(+) to Virus(−). Consequently, eight genes (C17orf80, SUB1, PYROXD2, IFT57, PAM, ARMC8, SSR3, and BNC2) were in common. The first five genes showed expression changes in the same direction with negative fold changes for C17orf80 and SUB1, and positive for PYROXD2, IFT57, and PAM in both Virus(+)-primary and -metastatic specimens (Additional file 9: Figure S8D). Since the primary tumor analysis involves various viruses, the 69 DEGs were further compared to the four genes that had differential expressions between Y73SV(+) and Y73SV(−) primary NSCLC tumors. No overlapping gene was found. Collectively, these observations suggested that Y73SV might have different host cellular gene targets and differently influence their expressions in primary and metastatic tumor cells.

## Discussion

Viruses are now accepted as bona fide etiologic factors of human cancer such as with HPV in cervical and oropharyngeal cancer, and HBV in hepatocellular carcinoma [1]. In our recent published study, we screened archived frozen NSCLC specimens and 10 non-neoplastic controls for potential microorganisms and surprisingly found most SCC and adenocarcinomas contained various strains of viral DNA, but they were absent in non-neoplastic lung. These data raised the question of whether viral DNA we found were just from commensal viruses that somehow were attracted to the tumors or whether they were functional and were in some way involved in carcinogenesis. In the current study, we thus analyzed gene expression microarray data to investigate the transcriptomic targets and differential gene expression patterns by virus infection status in these same NSCLC tumor specimens.

We showed that various genes, oncogenic gene sets, and immunologic gene sets had significantly altered gene expression profiles between Virus(+) and Virus(−) NSCLC tumors with the same histological subtype or metastatic disease status according to viral types. For example, the cell cycle, proteasome, and p53 signaling pathways were significantly over-represented biological processes among DEGs in primary NSCLC tumor microarray analysis. This finding of the cell cycle pathway is in parallel with a known cancer-related mechanism of transforming retroviruses which carry oncogenes derived from cellular genes that are involved in mitogenic signaling and growth control [48]. Furthermore, it was reported that a viral oncoprotein E6 in HPV induces proteasomal degradation of the tumor-suppressor p53, thereby compromising cell-cycle arrest and apoptosis [2].

One of most remarkable findings in our study was the presence of 22 viral carcinogenesis pathway-associated DEGs in primary NSCLC tumors. On the other hand, this pathway was not found when metastatic NSCLC specimens were analyzed solely or together with primary tumors, suggesting that these DEGs from our study are highly plausible transcriptomic targets of viruses for developing primary NSCLC and more extensive investigation of them will shed a light on delineating the potential etiological roles of viruses in NSCLC.

Notably, there was no overlap between the lists of DEGs from the analyses of each viral type that infected the primary NSCLC tumor specimens. Also, several significantly enriched hallmarks, oncogenic signatures, and immune-related signatures were identified in the analysis of SCC tumors, but none in the analysis of adenocarcinoma and BAC. These findings support that differential gene expression patterns of NSCLC tumors were quite distinct among infected viral types even in the same histological subtype.

We also found that eight DEGs were common in both primary tumor analysis and metastatic tumor analysis, and some of them even showed opposite directions of expression changes, in terms of different signs of fold changes. This implies that viruses may have the same host gene targets during NSCLC progression but might regulate their target gene expressions differently.

It is widely agreed that BLV does not cause disease in humans, but we found a high incidence (85%) of several Delta retroviruses in our lung SCC specimens, including BLV. We also identified several genes and gene pathways significantly altered between BLV-infected and uninfected lung SCC specimens. Recently, BLV was detected in a high proportion (59%) of 218 breast cancer specimens [49] and a statistical association between BLV and the risk of breast cancer was reported [50]. Although this virus has not yet been causally linked to cancer development or progression, it may play an important role that yet to be described.

In this study, we focused primarily on annotating biological functions and virus-host protein interactions of DEGs between virus-infected and uninfected NSCLC, but not on the association of those genes with clinical outcomes of patients. Our previous study [3] already documented that NSCLC patients whose tumor contained viral DNA had a better prognosis, longer overall survival times, than those without viral DNA in their tumors. The DEGs in these viral-positive tumors that are strongly associated with a more positive clinical outcome in NSCLC patients may serve as potential biomarkers or even novel therapeutic targets for treatment to improve outcomes.

In our NSCLC tumor specimens, particular viruses were detected in certain histologic subtypes and at a cancer progression stage (e.g. SCC tumors infected with BLV, PlpPV1, and STLV, and metastatic lung cancer with Y73SV, and primary NSCLC with HTLV-1). The cell types of lung cancer have far different presentations and usual anatomic location in the lungs: adenocarcinoma primarily starts in the periphery of the lungs and squamous cell usually arises in larger airways. There tend to be differences in the demographics of the patients who develop the different tumor cell types. For example, lung squamous cell carcinoma almost always occurs in current or former heavy smokers (and predominantly in men), whereas adenocarcinoma commonly occurs in former minimal smokers or never smokers. So it is not surprising that different types of viral DNA are found in different tumor cell types.

Since our current study has a small number of NSCLC tumors, we were not able to take account for interaction effects of viral types, histological subtypes, and metastatic disease sites on gene expression profiles in diverse NSCLC tumors. We also identified differentially altered genes and inferred their functional relationships with the detected viruses solely by a statistical comparison and bioinformatics approaches. A larger study comparing the impact of tumor viruses on gene expression in primary tumors to those in metastatic NSCLC will be needed in conjunction with a biological confirmation of expression changes of the genes and the presence of viral proteins by PCR and immunostaining experiments to better understand complex virus-host gene interactions in the NSCLC tumors.

## Conclusions

Differential gene expression patterns were distinct among infected viral types, histological subtypes, and metastatic disease status of NSCLC. These findings support the hypothesis that tumor viruses play a role in NSCLC by regulating host gene expressions in tumor cells during tumor differentiation and progression. Therefore this follow-up study strongly indicates that the viral DNA detected in lung tumors was from functional viruses interacting with tumor cells and they were not just “passengers,” a unique finding in lung carcinogenesis that warrants further investigation.

## Additional files

Additional file 1: **Figure S1.** Expression Patterns of 639 Genes Differentially Altered between all Virus-infected and Uninfected NSCLC Tumor Specimens. (PDF 163 kb)

Additional file 2: **Table S1.** Top 10 Significant Genes with Differential Expression between Virus-infected and uninfected NSCLC Tumor Specimens . (PDF 197 kb)

Additional file 3: **Figure S2.** Differentially-Expressed Genes between Virus-infected and Uninfected Primary NSCLC Tumors. (PDF 181 kb)

Additional file 4: **Figure S3.** Viral Carcinogenesis (KEGG ID: hsa05203). (PDF 134 kb)

Additional file 5: **Figure S4.** Gene Sets Enriched Significantly in All Primary NSCLC. (PDF 176 kb)

Additional file 6: **Figure S5.** Expression Patterns of 12 Genes Differentially Expressed between Virus-infected and Uninfected Squamous Cell Carcinoma. (PDF 137 kb)

Additional file 7: **Figure S6.** Differentially-Expressed Genes between Virus-infected and Uninfected Adenocarcinoma. (PDF 147 kb)

Additional file 8: **Figure S7.** Gene Expression of Notch-signaling and CRX_NRS Gene sets in Bronchioloalveolar Carcinoma. (PDF 148 kb)

Additional file 9: **Figure S8.** Differentially-Expressed Genes between Virus-infected and Uninfected Metastatic NSCLC. (PDF 129 kb)

## Abbreviations

BAC: Bronchioalveolar carcinoma
BLV: Bovine leukemia virus
DAVI: Database for annotation, visualization and integrated discovery
ES: Enrichment score
FDR: False discovery rate
GO: Gene ontology
GSEA: Gene set enrichment analysis
HBV: Hepatitis B virus
HPV: Human papilloma virus
HTLV-1: Human T-cell lymphotropic virus type I
LIMMA: Linear models for microarray analysis
LLMDA: Lawrence Livermore microbial detection array
NSCLC: Non-small cell lung cancer
PCR: Polymerase chain reaction
PCV-2: Porcine circovirus type 2
PlpPV1: *Panthera leo persica* papillomavirus type 1
SCC: Squamous cell carcinoma
STLV1: Simian T-cell leukemia virus type 1
STLV2: Simian T-cell leukemia virus type 2
STLV6: Simian T-cell leukemia virus type 6
Y73SV: Y73 sarcoma virus
