# Supplementary material for: Impact of viral presence in tumor on gene expression in non-small cell lung cancer
Source: BMC Cancer. 2018 Aug 22;18:843. doi: 10.1186/s12885-018-4748-0 (PMC6106745; doi:10.1186/s12885-018-4748-0)

Supplementary Figure 1. Expression Patterns of 639 Genes Differentially Altered Between Virus-infected (n=21) and Uninfected NSCLC Tumor Specimens (n=15)

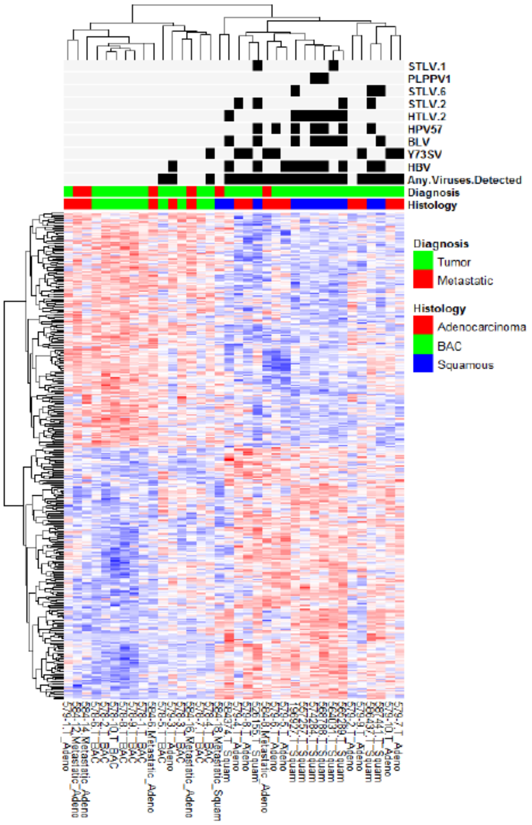

Supplement: Supplementary file 1 — Figure S1. Expression Patterns of 639 Genes Differentially Altered between all Virus-infected and Uninfected NSCLC Tumor Specimens. (PDF 163 kb) [file 12885_2018_4748_MOESM1_ESM.pdf]
