# Supplementary material for: Impact of viral presence in tumor on gene expression in non-small cell lung cancer
Source: BMC Cancer. 2018 Aug 22;18:843. doi: 10.1186/s12885-018-4748-0 (PMC6106745; doi:10.1186/s12885-018-4748-0)
Supplement: Supplementary file 2 — Table S1. Top 10 Significant Genes with Differential Expression between Virus-infected and uninfected NSCLC Tumor Specimens . (PDF 197 kb) [file 12885_2018_4748_MOESM2_ESM.pdf]

Supplementary Table 1. Top 10 Significant Genes with Differential Expression between Virus-infected and uninfected NSCLC Tumor Specimens

Row	Gene Symbol	Probe set	Mean In Virus (+)	Mean In Virus (-)	Fold Change (FC)	log_2 FC	Up/Down	P-value	FDR P-value	Gene Description
1	JMJD1C	merck-AK024991_at	279.6221	123.4899	0.441632	1.142613	down	1.96E-07	0.011902	jumonji domain containing 1C
2	PCYT1A	merck2-NM_005017_at	157.9334	354.177	2.242572	-1.17424	up	1.54E-06	0.031184	phosphate cytidylyltransferase 1, choline, alpha
3	CTNNB1	merck-AF086341_at	282.1095	129.4862	0.458993	1.109964	down	1.52E-06	0.031184	catenin beta 1
4	PCYT1A	merck-BM560359_a_at	124.4573	268.2678	2.155502	-1.12853	up	3.70E-06	0.037416	phosphate cytidylyltransferase 1, choline, alpha
5	UFD1L	merck2-AF141201_at	1877.233	2694.626	1.435424	-0.53107	up	3.01E-06	0.037416	ubiquitin fusion degradation 1 like (yeast)
6	AMN	merck-NM_030943_at	54.76712	31.08339	0.567556	0.778991	down	9.75E-06	0.073898	amnion associated transmembrane protein
7	XXYLT1	merck-NM_152531_at	289.0155	613.2811	2.121966	-1.03735	up	1.23E-05	0.082644	xyloside xylosyltransferase 1
8	GNB5	merck-CR624892_a_at	85.10777	154.6089	1.816625	-0.84122	up	1.93E-05	0.083851	G-protein subunit beta 5
9	OSTF1	merck-NM_012383_at	295.83	527.427	1.782872	-0.80764	up	2.35E-05	0.083851	osteoclast stimulating factor 1
10	E2F4	merck-NM_001950_at	450.4585	669.1157	1.48541	-0.57578	up	1.53E-05	0.083851	E2F transcription factor 4
