# Supplementary material for: Impact of viral presence in tumor on gene expression in non-small cell lung cancer
Source: BMC Cancer. 2018 Aug 22;18:843. doi: 10.1186/s12885-018-4748-0 (PMC6106745; doi:10.1186/s12885-018-4748-0)

Supplementary Figure 2. Differentially-Expressed Genes Between Virus-infected and Uninfected Primary NSCLC Tumors

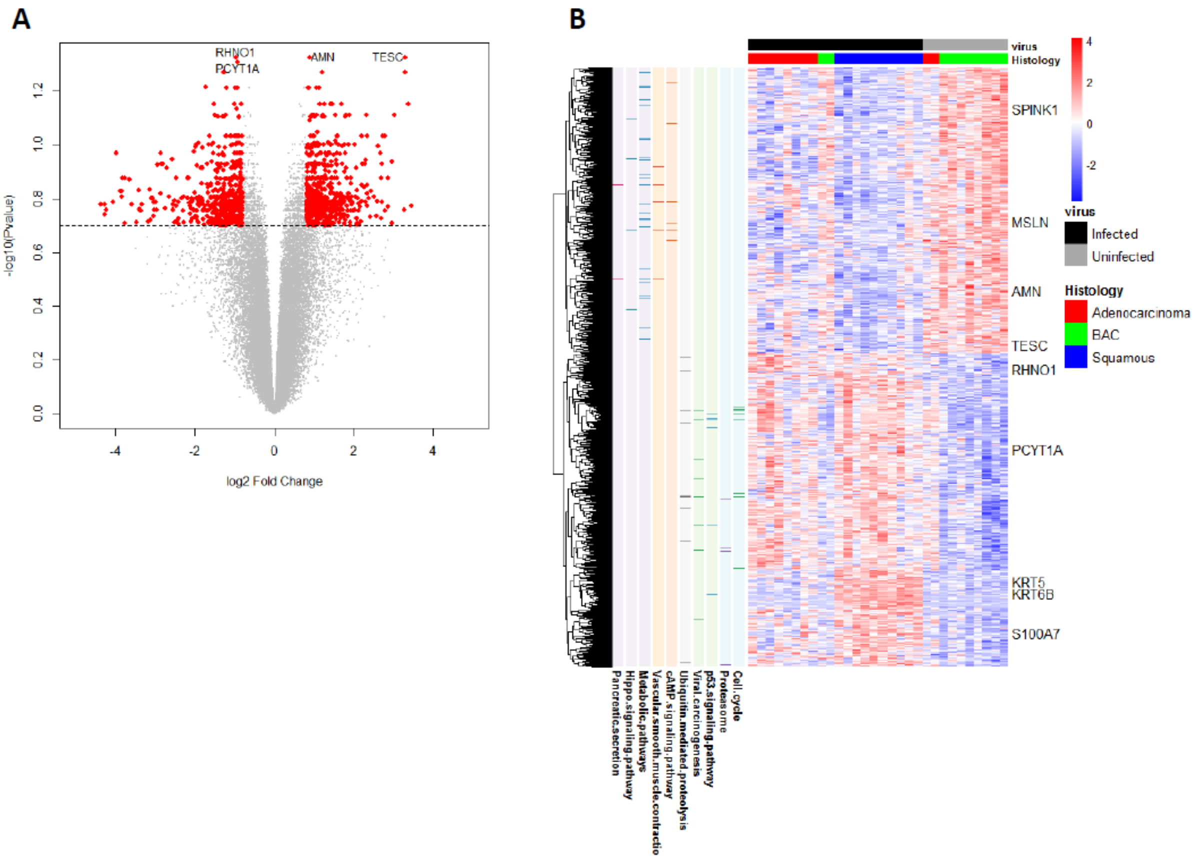

Supplement: Supplementary file 3 — Figure S2. Differentially-Expressed Genes between Virus-infected and Uninfected Primary NSCLC Tumors. (PDF 181 kb) [file 12885_2018_4748_MOESM3_ESM.pdf]
