# Supplementary figures and images for: Impact of viral presence in tumor on gene expression in non-small cell lung cancer
Source: BMC Cancer. 2018 Aug 22;18:843. doi: 10.1186/s12885-018-4748-0 (PMC6106745; doi:10.1186/s12885-018-4748-0)

**Supplementary Figure 3. Viral Carcinogenesis (KEGG ID: hsa05203)**

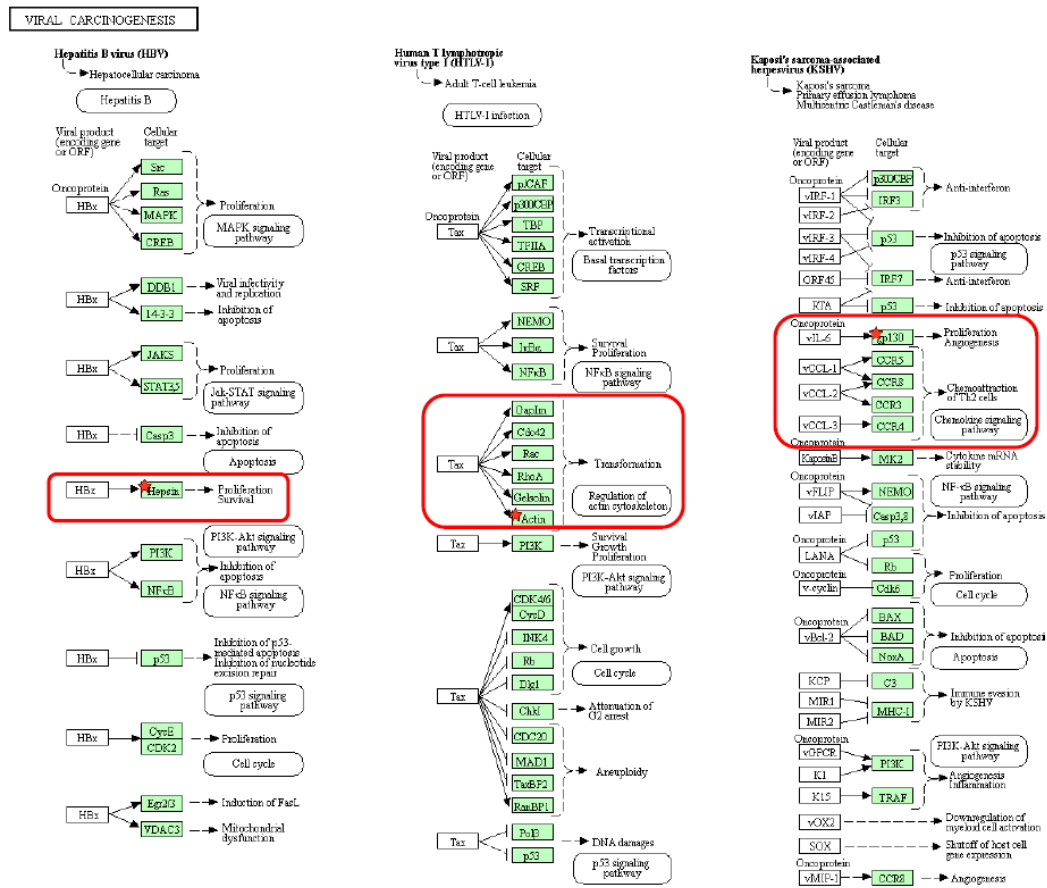

Supplement: Supplementary file 4 — Figure S3. Viral Carcinogenesis (KEGG ID: hsa05203). (PDF 134 kb) [file 12885_2018_4748_MOESM4_ESM.pdf]

Supplementary Figure 4. Gene Sets Enriched Significantly in All Primary NSCLC

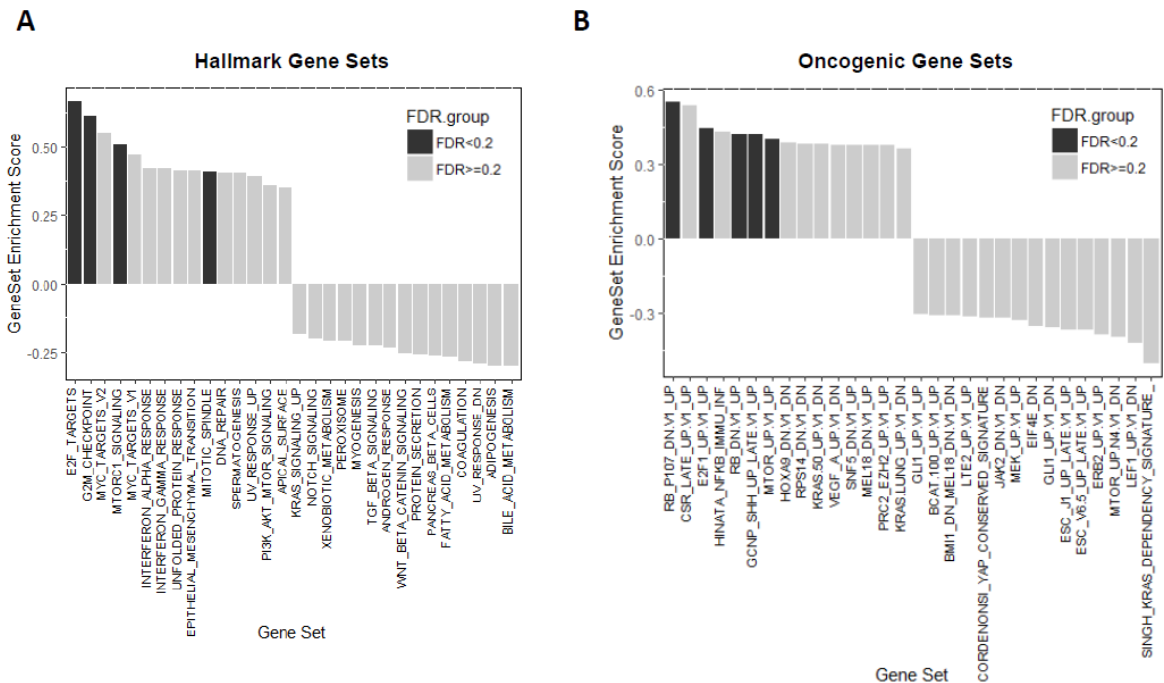

Supplement: Supplementary file 5 — Figure S4. Gene Sets Enriched Significantly in All Primary NSCLC. (PDF 176 kb) [file 12885_2018_4748_MOESM5_ESM.pdf]
