# Supplementary material for: Impact of viral presence in tumor on gene expression in non-small cell lung cancer
Source: BMC Cancer. 2018 Aug 22;18:843. doi: 10.1186/s12885-018-4748-0 (PMC6106745; doi:10.1186/s12885-018-4748-0)

Supplementary Figure 5. Expression Patterns of 12 Genes Differentially Expressed between Virus-infected and Uninfected Squamous Cell Carcinoma

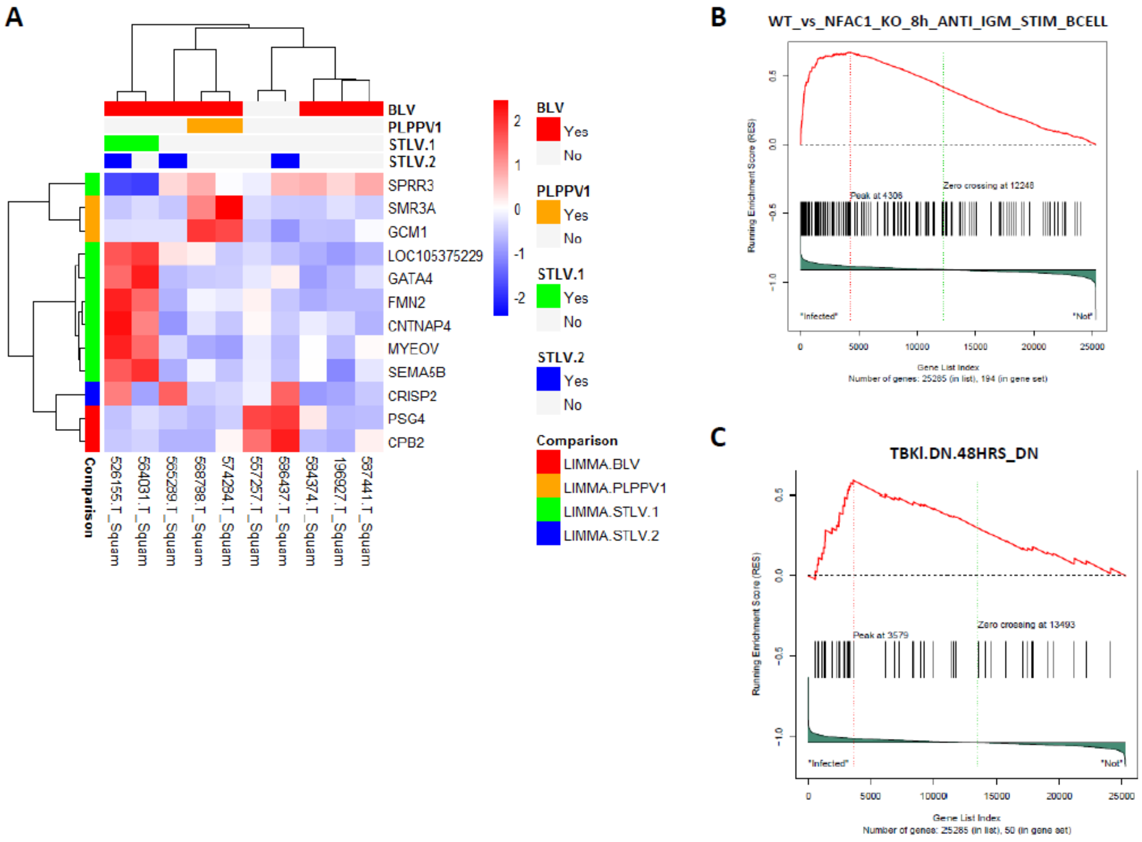

Supplement: Supplementary file 6 — Figure S5. Expression Patterns of 12 Genes Differentially Expressed between Virus-infected and Uninfected Squamous Cell Carcinoma. (PDF 137 kb) [file 12885_2018_4748_MOESM6_ESM.pdf]
