# Supplementary material for: Impact of viral presence in tumor on gene expression in non-small cell lung cancer
Source: BMC Cancer. 2018 Aug 22;18:843. doi: 10.1186/s12885-018-4748-0 (PMC6106745; doi:10.1186/s12885-018-4748-0)

Supplementary Figure 6. Differentially-Expressed Genes between Virus-infected (n=8) and Uninfected Adenocarcinoma (n=2)

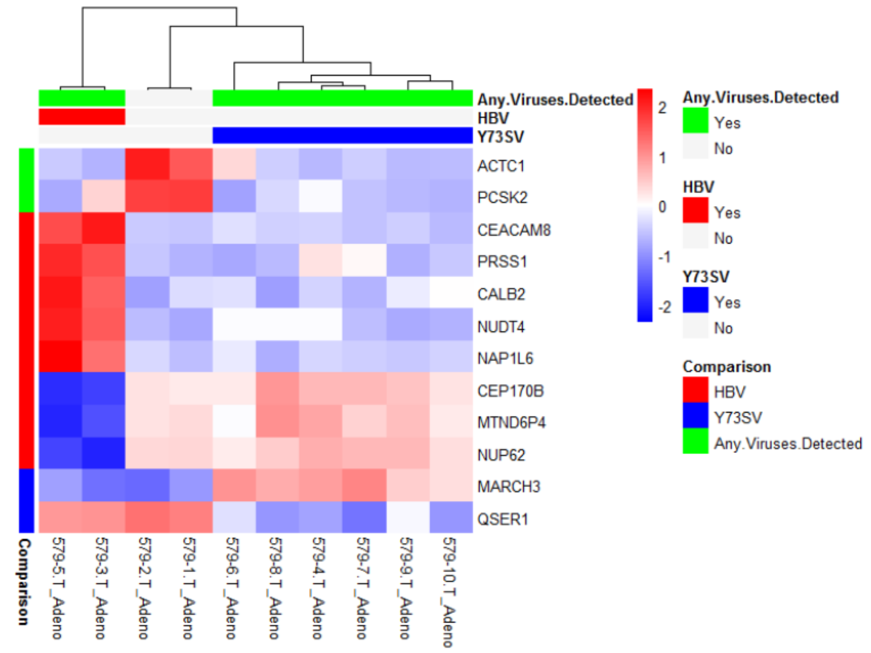

Supplement: Supplementary file 7 — Figure S6. Differentially-Expressed Genes between Virus-infected and Uninfected Adenocarcinoma. (PDF 147 kb) [file 12885_2018_4748_MOESM7_ESM.pdf]
