# Supplementary material for: Impact of viral presence in tumor on gene expression in non-small cell lung cancer
Source: BMC Cancer. 2018 Aug 22;18:843. doi: 10.1186/s12885-018-4748-0 (PMC6106745; doi:10.1186/s12885-018-4748-0)

Supplementary Figure 7. Differential Gene Expression Pattern of Bronchioloalveolar Carcinoma

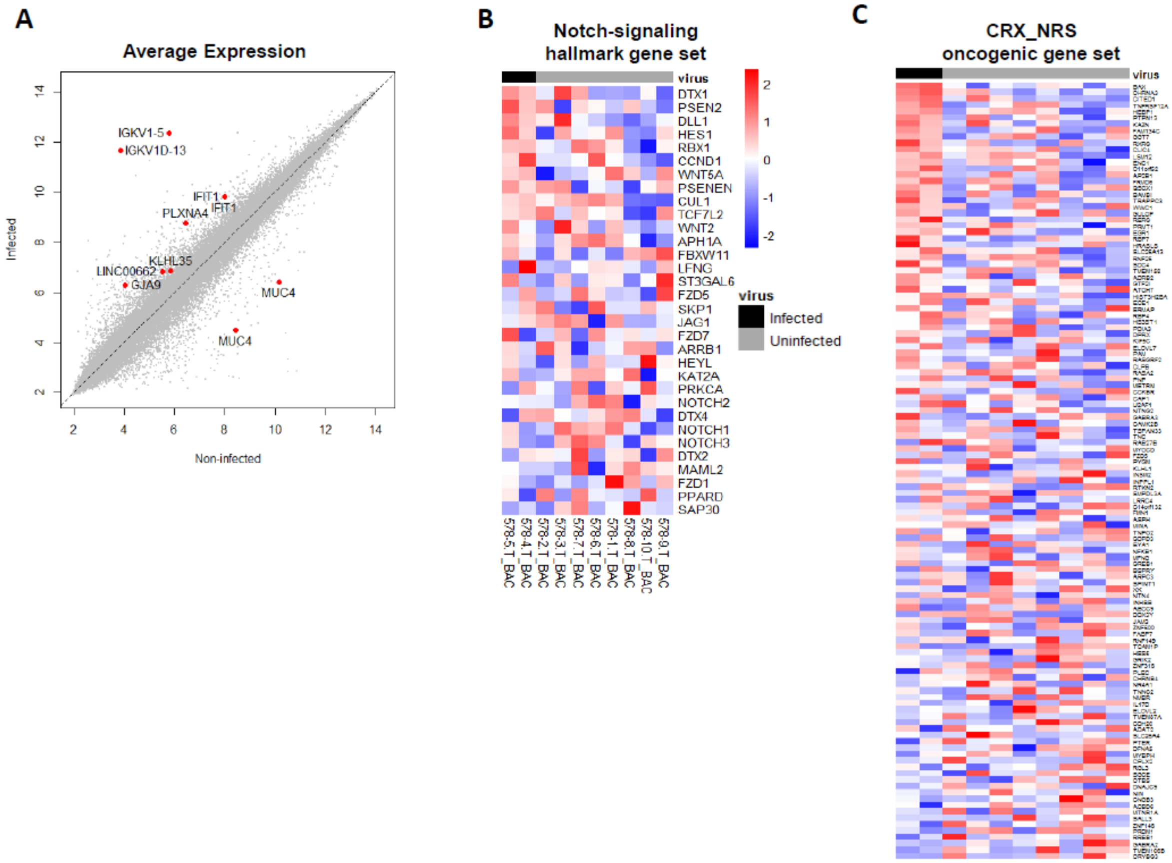

Supplement: Supplementary file 8 — Figure S7. Gene Expression of Notch-signaling and CRX_NRS Gene sets in Bronchioloalveolar Carcinoma. (PDF 148 kb) [file 12885_2018_4748_MOESM8_ESM.pdf]
